# Tapioca Dextrin as an Alternative Carrier in the Spray Drying of Fruit Juices—A Case Study of Chokeberry Powder

**DOI:** 10.3390/foods9081125

**Published:** 2020-08-15

**Authors:** Jolanta Gawałek, Ewa Domian

**Affiliations:** 1 Institute of Food Technology of Plant Origin, Poznań University of Life Sciences, Wojska Polskiego 31, 60-624 Poznań, Poland; 2 Department of Food Engineering and Process Management, Warsaw University of Life Sciences—SGGW, Nowoursynowska 159c, 02-776 Warsaw, Poland; ewa_domian@sggw.pl

**Keywords:** spray drying, fruit powders, chokeberry, tapioca dextrin carrier, bioactive compounds, antioxidants, phenolic content, physicochemical properties of powder

## Abstract

This paper analyses the semi-industrial process of spray drying chokeberry juice with carbohydrate polymers used as a carrier. Tapioca dextrin (Dx) was proposed and tested as an alternative carrier and it was compared with maltodextrin carriers (MDx), which are the most common in industrial practice. The influence of selected process parameters (carrier type and content, inlet air temperature, atomiser speed) on the characteristics of dried chokeberry powder was investigated. The size and microstructure of the powder particles, the bulk and apparent density, porosity, flowability, yield and bioactive properties were analysed. In comparison with MDx, the Dx carrier improved the handling properties, yield and bioactive properties. An increase in the Dx carrier content improved the phenolic content, antioxidant capacity, flowability and resulted in greater yield of the powder. An increase in the drying temperature increased the size of particles and improved powder flowability but it also caused a greater loss of the phenolic content and antioxidant capacity. The rotary atomizer speed had the most significant effect on the bioactive properties of obtained powders, which increased along with its growth. The following conditions were the most favourable for chokeberry juice with tapioca dextrin (Dx) as the carrier: inlet air temperature, 160 °C; rotary atomizer speed, 15,000 rpm; and Dx carrier content, 60%.

## 1. Introduction

The growing consumption of instant foods around the world causes an increasing demand for dried products. Consumers expect products that enable quick and easy preparation of meals and their storage [1]. An increasing demand for natural and health-promoting food is the second most important trend in the food industry [2]. Both trends have intensified in recent years and significantly increased the popularity of these products. Dried fruit products are ideal semi-finished goods that can be used in the production of this food. Both whole fruits and fruit pieces as well as pastes and juices can be dried [3]. Fruit powders match both of these food market trends, especially chokeberry powder, due to its properties. This product can be an excellent ingredient, as it colours, flavours and enriches food with bioactive substances. The effect is a health-promoting finished product.

Chokeberry (*Aronia melanocarpa* L.) is a fruit with various health-promoting properties. It prevents diseases of affluence, such as hypertension, atherosclerosis, diabetes and some types of cancer [4,5,6,7]. The consumption of chokeberry products delays ageing processes, prevents age-related eye diseases such as macular disorders and helps to maintain good night vision and visual acuity [8]. The skin maintains resistance to UV radiation [9]. Most of these properties of chokeberry result from its high content of antioxidants, which is 3–4 times greater than the content of antioxidants in blackberry, blackcurrant and raspberry [10,11,12]. Chokeberry fruits are one of the richest natural sources of anthocyanins (460–700 mg per 100 g) [12]. The food industry mostly offers chokeberry juice, which is not only a healthy product but also a natural red and blue pigment of great colouring strength because it is rich in anthocyanins [13]. Recently, the demand for chokeberry juice powder has increased due to the rising popularity of instant products and food for specified health use.

The spray drying of fruit juice is one of the most common methods of fruit powder production. The advantage of spray drying consists in the fact that it is a mild and fast dehydration process due to rapid evaporation. Changing the liquid form of juices into dry powders by reducing the water content prolongs the shelf life of products (decrease in water activity) [14]. It enables the maintenance of product stability and functionality until its use, such as reconstitution behaviour. It is not easy to dry fruit juice because it is sticky [15]. These properties are caused by the main ingredients of fruit juices, like low molecular weight sugars and organic acids, which are characterised by low glass transition temperature (Tg), high hygroscopicity, low melting temperature and high solubility in water. All these factors reduce product stability, yield (due to juice sticking to the dryer walls) and even cause difficulties operating the spray dryer [16]. Carriers, which are colourless and odourless high molecular weight additives, especially carbohydrate polymers such as hydrolysed starch and gum [17], are used to eliminate all these negative phenomena. Apart from carbohydrate polymers, proteins and lipids, such as whey proteins, soy proteins, sodium caseinate and lecithin could also be used as more effective carriers in the spray drying process [18,19], but they are more expensive. Therefore, cheaper carbohydrate polymers are usually chosen and their quantity is optimised. Potato and maize starches with various degree of saccharification are the most widely used carbohydrates for this purpose [20,21]. These low-priced mass-produced products can also efficiently stabilise pigments and other bioactive compounds by microencapsulation [18,22]. There are attempts to minimise the number of carriers added to industrially manufactured products because any amount that exceeds the required minimum may negatively affect the sensory quality of the product [23]. Therefore, researchers are trying to find optimal drying additives (carriers), the minimal addition of which will maintain the desired technological effect in the spray drying of fruit juices. Villacrez et al. [24] tested six different carbohydrate polymer carriers and three blends of these carriers in the spray drying of andes berry aqueous extract. Maltodextrin DE20 and Hi-Cap^TM^ 100 (chemically modified food starch refined from waxy maize) exhibited the highest retention of bioactive compounds (anthocyanins). They did not differ much in their effectiveness of stabilisation of bioactive compounds (<10%). Therefore, researchers continue looking for inexpensive carriers that will exhibit higher retention of bioactive compounds and other useful properties. If more efficient carriers are used for the production of powders with bioactive properties, they may be used in smaller quantities, which is very much expected by the food industry. However, as the economic aspect is a key issue in industrial practice, the price of new carriers cannot differ significantly from that of maltodextrin.

This study analyses the usefulness of tapioca dextrin as an alternative carrier to potato maltodextrin. Dextrins and maltodextrins are a class of low molecular weight carbohydrates resulting from partial acid and/or enzymatic hydrolysis of starch or glycogen. Maltodextrins are a combination of 3–20 D-glucose units that are primarily linked by α-1,4-bonds. Dextrins are saccharide polymers composed of D-glucose units linked primarily by α-1,4-bonds or α-1,6-bonds [25].

The functionality and quality of resulting powders are affected not only by the type of carrier used in the spray drying of fruit juices but also by the production process parameters, which influence their microstructure and properties [26,27,28]. Therefore, it is very important to choose the right parameters of the drying process to overcome operational difficulties (mostly juice sticking to the dryer walls during the process), which decrease the yield of powder [29]. Moreover, the food and pharmaceutical industries increasingly often seek more natural fruit powders with ultimate flavouring, colouring and bioactive properties, which can perfectly enrich end products. As powder made from spray-dried chokeberry juice has good handling and storage properties, it fits well with the current development trend in this branch of products. Therefore, it was reasonable to analyse the effectiveness of tapioca dextrin as a carrier in the spray drying of chokeberry juice and the influence of direct and indirect drying conditions on the properties of powders. The data from this study may be used for the spray drying of chokeberry juice in industrial production.

## 2. Materials and Methods

### 2.1. Materials

Chokeberry juice concentrate with 65° Brix extract content (SVZ International B. V., Breda, The Netherlands) was the research material. Tapioca dextrin (Dx) (trade name Crystal Tex 626, Ingredion) and for comparison—maltodextrin (MDx) with the saccharification degree (DE) = 8 (Roquette Freres) were used as carriers. Feed solutions with various carrier to chokeberry juice concentrate ratios were prepared for drying to make powders with 50, 60 and 70% content of the carrier in dry mass. Water was added to each solution to obtain 33% of dry mass. A system tested by Wesołowski and Gawałek was used for effective mixing and dissolution [30].

### 2.2. Spray Drying

The industrial drying process was fully simulated in a semi-industrial Niro Atomiser FU 11 DA spray dryer. Liquid was sprayed with a rotary atomiser. The dryer had pneumatic hammers, which periodically removed the residual powder from its walls. There were three different temperatures of inlet air in the drying process: 150, 160 and 170 °C, and three different speeds of the rotary atomiser: 12,000, 13,500 and 15,000 rpm. The process was conducted at variable inlet air temperatures and a rotary atomiser speed of 12,000 rpm, or at variable rotary atomiser speeds and an inlet air temperature of 160 °C. The outlet air temperature in all experiments was held at 88–90 °C by setting the raw solution flow between 10 and 15 L/h. The same method of collecting the product and dryer wall cleaning (pneumatic hammers impact frequency) was used in all the drying processes.

### 2.3. Particle Size Distribution

After dispersing the powders in isopropanol the size of particles was measured with a Cilas 1190 laser particle size analyser (Orleans, France). The results were shown as the 10th, 50th (median), and 90th percentile of the volume distribution of particle size.

### 2.4. Microstructure of Particles

The morphology of chokeberry juice powder was observed with a scanning electron microscope (Hitachi TM3000, Tokyo, Japan). The powders were gold sputtered (Cressington 108, UK).

### 2.5. Bulk Density, Flowability and Moisture Content

Loose bulk density (*ρ*_L_) and tapped bulk density (*ρ*_T100_ and *ρ*_T500_) (the bulk density of the powder packed with 100 and 500 standard taps) were measured with a STAV 2003 jolting volumeter (Engelsmann AG, Ludwigshafen, Germany). The flowability of powder was expressed as the Hausner ratio (HR), the loose and tapped bulk density HR_100_ = *ρ*_T100_/*ρ*_L_ and HR_500_ = *ρ*_T500_/*ρ*_L_ [31]. The moisture content of powder was analysed by the oven method at 105 °C for 4 h.

### 2.6. Apparent Particle Density and Bulk Porosity Powder Bed

The apparent particle density (*ρ*) was measured with a Stereopycnometer helium pycnometer (Quantachrome Instruments, Boynton Beach, FL, USA). The bulk porosity powder estimated from interstitial air in loose (ε_L_) or tapped powder bed (ε_T100_ and ε_T500_) was calculated from the apparent particle density (*ρ*) and bulk densities (*ρ*_L_, *ρ*_T100_, *ρ*_T500_) respectively, as follows: ε_L_ = 1 − *ρ*_L_/*ρ*, ε_T100_ = 1 − *ρ*_T100_/*ρ* and ε_T500_ = 1 − *ρ*_T500_/*ρ*.

### 2.7. Powder Yield

The following formula was used to calculate the powder yield after spray drying:

(1) $$Y=\frac{m_{p}\cdot w_{dmp}}{m_{f}\cdot w_{dmf}}\cdot 100\;\left[\%\right]$$

where

*m_p_, m_f_*—mass of powder and feed solution; kg_p_, kg_f_, respectively;
*w_dmp_, w_dmf_*—dry matter content in the powder and feed solution; kg*_dmp_*/kg*_p_*, kg*_dmf_*/kg*_f_*, respectively.

### 2.8. Total Polyphenol Content (TPC)

The Folin–Ciocalteu reagent was used to determine the total polyphenol content (TPC). Measurements were made with a Jasco V630 spectrophotometer (Tokyo, Japan) at a wavelength of 765 nm, according to the methodology used in our previous study [32]. Gallic acid was used as standard and the results were expressed as mg of gallic acid (GA) per 100 g d.m. (dry matter).

### 2.9. Antioxidant Capacity

The trolox equivalent antioxidant capacity (TEAC) was measured with the ABTS method, by means of a Jasco V630 spectrophotometer (Japan), in accordance to the methodology described by Re et al. [33]. A sample (30 μL of solution: 0.5 g of powder in 10 mL of 70% (*v*/*v*) acetone/water solution) was mixed with 3 mL of ABTS^+^ solution (after incubation with K_2_S_2_O_8_). The reaction was carried out in darkness for 6 min. Next, the absorbance values were measured at 734 nm. The results were expressed as mg trolox equivalents (TE) per 100 g d.m.

### 2.10. Statistical Analysis

There were at least 3 replicates of all physicochemical measurements. The hypothesis about the significant effect of the inlet air temperature, rotary atomiser speed, carrier content and type (Dx or MDx) on the values of the parameters under analysis was verified by means of one- and two-way analysis of variance (ANOVA) with the Statgraphics 12 program. Differences between the mean values were evaluated with Tukey’s test at a significance level of α = 0.05. The homogenous groups of mean values were marked with letters.

## 3. Results and Discussion

### 3.1. Particle Size

The size of dried fruit juice particles obtained in the spray drying process influences physical properties, such as bulk density, flowability, compressibility, solubility and hygroscopicity [34]. Table 1 shows that the range of particle sizes of spray-dried chokeberry juice was narrower and had a relatively uniform distribution. The span value expressing the width of the particle size distribution was at the same low level for all powders (1.219–1.298). The mean diameter of particles (median D_50_) ranged from 39 to 50 μm (Table 1). The substitute diameters D_10_ and D_90_ ranged from 14 to 20 μm and 64 to 82 μm, respectively (Table 1).

The increase in the inlet air temperature caused an increase in all equivalent diameter values (D_10_, D_50_, D_90_) in the upper range of tested temperatures (T = 170 °C). The analysis of variance did not show any statistically significant changes in other ranges. The changes were caused by an increase in the energy delivered to the sprayed juice droplets and an increase in the drying rate, i.e., a higher drying rate reduced the shrinkage of particles [35]. Gawałek et al. [32], Bednarska and Janiszewska-Turak [36] also observed this trend in chokeberry juice dried on a maltodextrin carrier (MDx). Other authors also noted this dependency when drying other fruit juices: acai juice [37], jamun fruit juice [38], guava juice [39], tamarind pulp [40]. An increase in the rotary atomiser speed decreased the size of droplets in the solution. As a result, the powder particles were smaller. These dependencies were also observed by Chegini and Ghobadian [41] in orange juice and by Gawałek et al. [32] in chokeberry juice dried with a maltodextrin carrier (MDx). There were no statistically significant changes observed in chokeberry juice dried with the tapioca dextrin carrier for all equivalent diameters (Table 1).

The ANOVA proved that the drying process with the Dx carrier resulted in larger particles than the process with the MDx carrier (Table 1). This effect may have been caused by the higher feed solution viscosity of the Dx carrier. The spray drying of more viscous solutions usually results in larger particles of powder [42]. The statistical analysis proved significant changes for different content of both carriers—MDx and Dx. In both cases the lowest values were observed for the medium content of 60%, while the highest values were observed for the lowest carrier content, i.e., 50%.

### 3.2. Microstructure of Powder Particles

**Figure 1:**
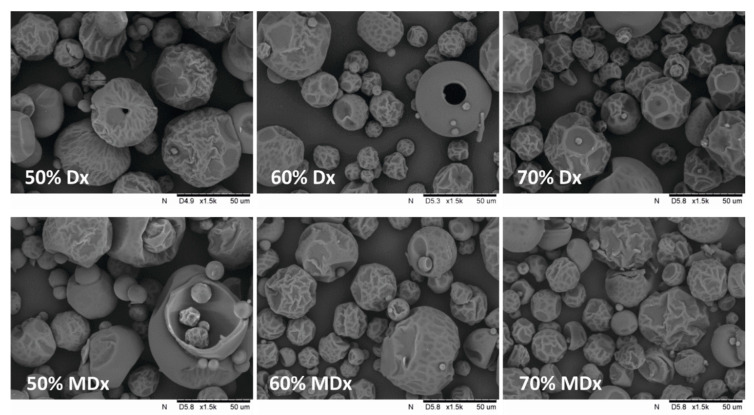
Microscopic images of spray-dried chokeberry juice particles for various types and content of the carrier at 1500x magnifications (Dx—tapioca dextrin, MDx—potato maltodextrin).

The particles produced in the spray drying process may differ in size, morphology and structure [23]. Figure 1 shows micrographs of powder particles with different type and mass fraction of the wall material (carrier). Spray drying very often results in hollow particles, which favour the formation of cracks and wrinkles on their surface, because air is trapped inside while particles are being formed. If feed has been prepared using a high shear mixing process, air in the form of tiny bubbles is entrained into the liquid.

The resulting particles had various sizes and spherical shapes with multiple creases and cracks on their surfaces. As the content of both carriers (Dx and MDx50–70% d.m.) decreased, the number of creases and other deformations increased. When the content of simple sugar increases, particles tend to swell due to a smaller amount of the carrier added [32]. The Dx carrier resulted in fewer deformations and shrinkages on the surface of particles than the MDx carrier. Other authors also observed similar images of the microstructure of dried fruit juice particles on maltodextrin carriers [32,36,43,44,45].

### 3.3. Bulk Density, Flowability and Moisture Content

Bulk density is one of the most important properties of powder for its storage, processing, packaging and distribution [46]. It is a very complex property, which results from several other properties. However, the primary determinants of bulk density are particle density, the amount of interstitial air (i.e., external porosity), and flowability [47]. Powders obtained as a results of the spray drying of fruit juice usually have a wide range of particle sizes, which means that small particles can closely fill the spaces between large ones. Consequently, these powders contain a minimal volume of interstitial air [48], which results in a relatively high bulk density. In this study, the loose and tapped bulk densities of chokeberry juice powders were *ρ_L_* 520–594, *ρ_T100_* 598–697 and *ρ_T500_* 735–764 kg/m^3^, respectively (Table 2). There were statistically significant changes in bulk density of the powder with the Dx carrier when the inlet air temperature changed. An increase in the temperature (150–170 °C) caused a 14% increase in the loose bulk density and 6.2% and 3.9% increases in the tapped bulk density. The spray drying of chokeberry juice on an MDx carrier [32] as well as the spray drying of other fruit juices resulted in a completely opposite relationship between temperature and bulk density [49,50,51]. However, Zareifard et al. [52] and Mishra et al. [44] also noted a growing dependence in the experiments which they conducted respectively on lime and hog plum juice dried with maltodextrin.

Santhalakshmy et al. [38] conducted research on jamun fruit dried with maltodextrin and observed statistically insignificant changes in bulk and tapped density, depending on the inlet air temperature. The statistical analysis of the effect of the carrier content on loose bulk density *ρ*_L_ showed no significant changes when the Dx carrier was used, but the MDx carrier resulted in a decreasing trend. The tapped bulk density *ρ*_T100_ and *ρ*_T500_ gave the opposite results. Its value decreased while the content of the Dx carrier increased. When the MDx carrier was used, changes in the *ρ*_T500_ values were statistically insignificant. The relation observed with the MDx carrier was consistent with the findings of the study by Gawałek et al. [32]. There were similar results reported by Goula and Adamopoulos [50] (orange juice with maltodextrin), Zareifard et al. [52] (lime juice with maltodextrin), Patil et al. [53] (guava juice with maltodextrin) and Mishra et al. [44] (hog plum juice with maltodextrin).

Table 2 shows the values of the Hausner ratio for powders with different bed packing degrees (HR_100_ and HR_500_). According to de Jong et al. [31], powders with an HR of 1–1.25, 1.25–1.4 and >1.4 are free flowing, easy flowing and difficult flowing, respectively. According to Hayes [54], powders with an HR of 1–1.1, 1.1–1.25, 1.25–1.4 and >1.4 are free flowing, medium flowing, difficult flowing and very difficult flowing, respectively. In our study the flowability of the chokeberry powders deteriorated as the bed packing degree increased.

In the state of slight packing (HR_100_ = 1.11–1.27) the powders could be classified as medium flowing, whereas in the packed state (HR_500_ = 1.35–1.42) the same powders might be classified as difficult flowing (according to the Hayes scale). The reduced material flowability, which was observed in our study along with the increase in the bed packing of fine-grained powders, is consistent with other authors’ findings [46,55]. It is caused by increased cohesion of powders in the packed state. The analysis of the effect of inlet air temperature on the flowability of powders with the Dx carrier showed that this property improved significantly at the highest temperature (T = 170 °C). The same correlation was observed by Gawałek et al. [32] in an experiment on chokeberry juice dried with the MDx carrier. This dependence correlates with the particle size and it is the greatest at T = 170 °C. Improved flowability, which was observed along with the increase in the size of particles as a result of a change in the operating conditions, is in line with the observations of other authors, who used the Hausner ratio to evaluate the flowability of different types of powders [55,56]. As far as the rotary atomiser speed of 12,000–15,000 rpm is concerned, there were no statistically significant changes observed for the Dx carrier, as opposed to the MDx carrier [32]. In both cases, the increase in the content of the carrier caused a slight decrease in the Hausner ratios of HR_100_ and HR_500_, but the biggest drop was observed for MDx in the state of slight packing (HR_100_). Moreira et al. [57] found a similar correlation in a study on the drying of acerola pomace juice.

All the powders had similar average moisture content, i.e., from 2.31 ± 0.32% wb to 2.85 ± 0.26% wb.

### 3.4. Apparent Particle Density and Bulk Porosity Powder Bed

The apparent particle density *ρ* of the powders in our study ranged from 1290 to 1381 kg/m^3^ (Table 2). Both carriers (Dx and MDx) caused a statistically significant drop in the *ρ* value when their content increased. The Dx carrier resulted in respectively lower apparent particle density values, as the two-way analysis of variance proved. Patil et al. [53] noted similar results when they dried guava pulp using maltodextrin as the carrier.

External porosity is a very complex property. It is determined by the distribution of particle sizes, the shape of particles and the degree of agglomeration [55]. The bulk porosity powder bed ε_L_ of the chokeberry powders ranged from 0.393 to 0.445 (Table 2). The statistical analysis showed a significant increase in porosity with the highest content (70%) of both the Dx and MDx carriers. The two-way analysis of variance proved that the carrier type had no effect on the porosity of powders. However, the analysis of the drying process conditions where the Dx carrier was used showed significant influence of the inlet air temperature and the rotary atomiser speed. In both cases an increasing dependency could be seen. As far as the rotary atomiser speed is concerned, the powder bed porosity increased significantly at 15,000 rpm.

### 3.5. Powder Yield

Powder yield is one of the most important parameters in industrial practice because it directly affects the economic aspect of the process. Table 3 shows the yield of chokeberry powder *Y* under various drying conditions. The values ranged from 25.4% to 80.1% for the MDx carrier and from 35.3% to 95.3% for the Dx carrier. Both carriers caused a statistically significant increase in the powder yield when their content increased. However, there were higher values of this parameter for particular carrier contents when Dx was used. The lowest content (50%) of both the MDx and Dx carriers caused a very high drop in the yield. This fact may lead to the conclusion that the industrial production of chokeberry powder with such a low carrier content is economically unjustified. When the carrier content amounted to at least 60%, the yield of both carriers in our study ranged from 74.3% to 95.3%. It guarantees an acceptable loss level in industrial production. The results show that the Dx carrier gave a 10–15% higher yield than the MDx carrier. The growing effect of the MDx carrier content on the powder yield was also observed by Vardin and Yasar [58] on pomegranate juice, Zareifard et al. [52] on lime juice, and by Troya et al. [59] on banana passionfruit pulp. Only Zareifard et al. [52] used a similar carrier content as we did in our study (applicable on an industrial scale) and they also observed a big difference in the powder yield between 50% and 60% of the carrier content. The absolute values of the powder yield cannot be directly compared because Zareifard et al. [52] used a product discharged through the bottom outlet of the cyclone and collected from the main chamber of the spray dryer to calculate the yield. Our study was conducted on a semi-industrial scale. The conditions of the industrial process were simulated, including the use of impact hammers. Only the product discharged through the bottom outlet of the cyclone was included in the calculation of the yield.

The analysis of the effect of drying parameters on the yield of powder with the Dx carrier showed a statistically significant dependency for the drying temperature and an insignificant dependency for the rotary atomiser speed. An increase in the inlet air temperature caused a higher yield. This trend corresponds with the powder flowability correlation (the powder flowability increased along with the drying temperature). There were analogical correlations observed for both drying parameters during the drying of chokeberry juice on the MDx carrier [32] and during the drying of other fruit juices such as: lime juice [52], jamun fruit juice [38], tamarind pulp [40], black mulberry [49], banana passionfruit pulp [59]. Chegini and Ghobadian [60] used a small laboratory dryer without a regular wall residue brushing system in their experiment on the drying of orange juice. They observed the opposite dependency—higher temperatures reduced the yield.

### 3.6. Total Polyphenol Content (TPC)

A high content of polyphenols in fruit powders is very desirable due to their strong colouring and antioxidative properties. Chokeberry is classified as a fruit with a high content of polyphenols. Due to this fact it is used in the food industry as a natural colouring agent and as functional food [61,62]. However, spray-dried chokeberry powder with a high content of polyphenols is very desirable for instant food production. In this study the content of polyphenols (TPC) in the chokeberry powders and the TPC retention level (R_TPC_) was measured in relation to the initial content in the solutions before drying. The TPC in the powders obtained with the Dx carrier ranged from 1549 to 2014 mg GA/100 g d.m., whereas the retention ranged from 77.5% to 99.3%. The same parameters in the powders obtained with MDx ranged from 1309 to 1853 mg GA/100 g d.m. and from 71.3% to 83%, respectively (Table 3). The statistical analysis showed significantly higher values of the TPC and R_TPC_ for the powders made with the Dx carrier. The content of both the Dx and MDx carriers had analogical influence on the TPC and R_TPC_ values. An increase in the carrier content caused a statistically significant drop in the TPC (it was obvious due to the composition of powders). The opposite correlation was observed for the R_TPC_. This trend was caused by the higher content of the carrier, which provided better protection against high drying temperature and reduced the deposition/sticking of powder inside the drying equipment. Longer exposure and contact between powder and hot air, caused by the deposition of the drying material inside the installation between the impacts of a pneumatic hammer, has negative effect on polyphenolic compounds. The same effect of the carrier content on the degree of retention of polyphenols was observed by Gawałek et al. [32] in their research on chokeberry powders on the MDx carrier. Mishra et al. [44] and Moreno et al. [19] conducted experiments on the spray drying of hog plum juice and grape marc extract, respectively, and they made similar observations.

The inlet air temperature had a statistically significant effect on the phenolic content. Higher temperature resulted in a decrease in the TPC and R_TPC_ due to the susceptibility of polyphenols to thermal degradation. This trend was observed in the spray drying process of chokeberry powder with both the Dx and MDx carriers [32]. Ramírez et al. [63] studied passion fruit, blackberry, and mango juice, whereas Moreno et al. [19] tested grape marc extract and they reported similar results. However, the opposite trend was observed in the atomiser speed. Higher speed resulted in an increase in the content of polyphenols in the chokeberry powder. This effect was probably caused by the fact that bigger particles were formed at a lower speed of the atomiser and they tended to stick to the dryer walls. For this reason, the exposure time to the stream of hot air was extended until the impact of the pneumatic hammer detached residual powder from the dryer wall. Consequently, it increased the thermal degradation of bioactive substances.

### 3.7. Antioxidant Capacity

The use of the Dx carrier also resulted in a statistically higher antioxidant capacity expressed as TEAC and its retention R_TEAC_ in the chokeberry powders than the use of the MDx carrier. The TEAC values ranged from 13.3 to 17.1 mg TE/100 g d.m. for the Dx carrier and from 7.8 to 14.7 mg TE/100 g d.m. for the MDx carrier, whereas the R_TEAC_ values amounted to 60.6–86.9% for the Dx carrier and 30.6–75.5% for the MDx carrier (Table 3). The nature of changes was different than in the case of TPC. The highest TEAC values were noted for the 60% content of both the Dx and MDx carriers. The R_TEAC_ retention values were statistically lower for the lowest carrier content (50%). There were no statistically significant changes observed for the carrier content of 60% and 70%. There is no doubt that when the carrier content was close to 50%, it was too low to effectively secure the bioactive properties of chokeberry from the negative effect of temperature. Nambiar et al. [64] conducted laboratory experiments on tender coconut water and the MDx carrier at amounts of 10%, 30% and 50% and they found similar dependencies. The highest TEAC values were also obtained at the medium amount (30%). The tested range of the carrier content did not include the commercially effective range, because the powder yield in our study ranged from 9.37% to 16.04%. The loss of the antioxidant potential generated during the spray drying process was much greater than the loss of polyphenol content (TPC).

The inlet air temperature affected the TEAC and R_TEAC_ values in the chokeberry powders with the Dx carrier differently than the TPC values. The highest values were noted at 160 °C, the lowest—at 170 °C. This variability was probably caused by the fact that at the highest temperature (170 °C) massive thermal degradation of bioactive substances was observed due to the higher temperature of the drying air, whereas at the lowest temperature particles of powder were more likely to stick to the dryer walls, thus increasing the exposure time and TEAC loss. There were similar results reported by Silva et al. [65], who also observed the highest TEAC at a temperature of 160 °C (the researchers used the following inlet drying temperatures: 140, 160 and 180 °C). The statistical analysis of the influence of the rotary atomiser speed on the TEAC and R_TEAC_ showed that these values decreased significantly at the lowest speed, i.e., 12,000 rpm. The nature of this change was probably similar to the one described for the TPC.

## 4. Conclusions

The use of tapioca dextrin Dx as a carrier to dry the chokeberry juice concentrate resulted in a powder with better functional properties than the powders obtained with potato maltodextrin as a carrier, which is commonly used in the spray drying process. The growth of particles and moderate increase in the flowability of the powders as well as reduced surface deformation and shrinkage during drying resulted in the final product with better properties. In comparison to the MDx carrier, the Dx carrier changed the product yield significantly (the same amounts of both carriers were used). This aspect is very important in industrial practice because it can reduce the production loss. The Dx carrier also improved the retention of bioactive properties of the powders, which was manifested by the values of the polyphenols content (TPC) and antioxidant potential (TEAC).

The drying conditions affected the properties of the chokeberry powders dried with the Dx carrier. The research showed that the best conditions of the semi-industrial drying of chokeberry juice with the tapioca dextrin carrier Dx were: inlet air temperature, 160 °C; rotary atomiser speed, 15,000 rpm; and Dx carrier content, 60%.

Tapioca dextrin has great potential to become an effective carrier in the spray drying of chokeberry and other fruit juices, preserving their rich bioactive properties. As the properties of the powders obtained with the Dx carrier were better than those obtained with MDx, the carrier content can be decreased in industrial practice. The chokeberry powders obtained with 70% MDx and 60% Dx carrier content were characterised by comparable performance and retention of bioactive properties. Therefore, the use of Dx carrier could also make it possible to offer fruit powders with higher fruit content on the market.

## Figures and Tables

**Table 1:** Particle size distribution (D_10_, D_50_, D_90_, span) of spray-dried chokeberry powders.

Drying Conditions/Carrier Type and Content	D_10_ (μm)	D_50_ (μm)	D_90_ (μm)	Span
Dx, T = 150 °C	16.3 ± 0.5 ^a^	39.6 ± 0.8 ^a^	64.5 ± 1.9 ^a^	1.22 ± 0.01 ^a^
Dx, T = 160 °C	15.7 ± 0.7 ^a^	41.7 ± 1.4 ^a^	69.3 ± 2.5 ^a^	1.29 ± 0.00 ^b^
Dx, T = 170 °C	18.6 ± 0.9 ^b^	48.4 ± 1.1 ^b^	81.5 ± 2.9 ^b^	1.30 ± 0.01 ^b^
Dx, *n* = 12,000 rpm	15.7 ± 0.7 ^a^	41.7 ± 1.4 ^a^	69.3 ± 2.5 ^a^	1.30 ± 0.00 ^b^
Dx, *n* = 13,500 rpm	15.8 ± 0.4 ^a^	41.8 ± 0.5 ^a^	68.3 ± 1.3 ^a^	1.26 ± 0.01 ^a^
Dx, *n* = 15,000 rpm	14.9 ± 0.5 ^a^	40.2 ± 0.6 ^a^	65.9 ± 1.8 ^a^	1.27 ± 0.01 ^ab^
50% Dx	19.4 ± 1.1 ^D^	49.4 ± 1.3 ^C^	81.6 ± 3.4 ^C^	1.26 ± 0.02 ^AB^
60% Dx	15.7 ± 0.7 ^AB^	41.7 ± 1.4 ^AB^	69.3 ± 2.5 ^AB^	1.29 ± 0.00 ^BC^
70% Dx	17.8 ± 0.5 ^CD^	47.9 ± 0.8 ^C^	78.6 ± 1.9 ^C^	1.28 ± 0.01 ^ABC^
50% MDx	16.7 ± 0.7 ^BC^	43.8 ± 0.9 ^B^	71.6 ± 1.9 ^B^	1.25 ± 0.01 ^A^
60% MDx	14.3 ± 0.3 ^A^	39.2 ± 0.6 ^A^	64.2 ± 1.7 ^A^	1.27 ± 0.02 ^ABC^
70% MDx	14.9 ± 0.3 ^A^	41.9 ± 0.5 ^A^	69.3 ± 1.2 ^AB^	1.30 ± 0.01 ^C^

Dx—tapioca dextrin used as a carrier, MDx—potato maltodextrin used as a carrier, T—inlet drying air temperature, *n*—rotary atomizer speed, ^a, b^ Homologous groups for one of the factors: T, n, ^A, B, C, D^ Homologous groups of factors: carrier type and content.

**Table 2:** Loose bulk density (*ρ*_L_), tapped bulk density (*ρ*_T100_ and *ρ*_T500_), Hausner ratio (HR_100_ and HR_500_) as a flowability, apparent density (ρ) and bulk porosity of loose powder bed (ε_L_).

Drying Conditions/Carrier Type and Content	*ρ*_L_ (kg/m^3^)	*ρ*_T100_ (kg/m^3^)	*ρ*_T500_ (kg/m^3^)	HR_100_ (-)	HR_500_ (-)	*ρ* (kg/m^3^)	ε_L_ (-)
Dx, T = 150 °C	520.7 ± 3.8 ^a^	639.5 ± 4.1 ^a^	735.4 ± 3.4 ^a^	1.23 ± 0.01 ^b^	1.41 ± 0.02 ^b^	1325.2 ± 2.1^a^	0.39 ± 0.00^a^
Dx, T = 160 °C	546.2 ± 2.1 ^b^	662.8 ± 4.2 ^b^	756.3 ± 2.6 ^b^	1.21 ± 0.01 ^b^	1.38 ± 0.01 ^b^	1343.3 ± 0.4^c^	0.41 ± 0.00^b^
Dx, T = 170 °C	593.4 ± 2.3 ^c^	679.2 ± 2.7 ^c^	764.1 ± 1.7 ^c^	1.14 ± 0.02 ^a^	1.29 ± 0.01 ^a^	1333.8 ± 0.7^b^	0.45 ± 0.00^c^
Dx, *n* = 12,000 rpm	546.2 ± 2.1 ^b^	662.8 ± 4.2 ^b^	756.3 ± 2.6 ^b^	1.21 ± 0.01 ^a^	1.38 ± 0.01 ^ab^	1343.3 ± 0.4^c^	0.41 ± 0.00^a^
Dx, *n* = 13,500 rpm	522.9 ± 3.7 ^a^	641.1 ± 2.7 ^a^	737.8 ± 3.7 ^a^	1.23 ± 0.03 ^a^	1.41 ± 0.02 ^b^	1290.7 ± 0.4^a^	0.41 ± 0.00^a^
Dx, *n* = 15,000 rpm	545.2 ± 4.9 ^b^	648.2 ± 4.5 ^a^	738.4 ± 5.2 ^a^	1.19 ± 0.01 ^a^	1.35 ± 0.02 ^a^	1310.9 ± 1.0^b^	0.42 ± 0.00^b^
50% Dx	549.2 ± 7.8 ^AB^	697.0 ± 9.8 ^C^	777.4 ± 7.5 ^C^	1.27 ± 0.02 ^C^	1.42 ± 0.03 ^B^	1357.0 ± 0.3^D^	0.41 ± 0.01^A^
60% Dx	546.2 ± 2.1 ^AB^	662.8 ± 4.2 ^B^	756.3 ± 2.6 ^B^	1.21 ± 0.01 ^B^	1.38 ± 0.01 ^AB^	1343.3 ± 0.4^C^	0.41 ± 0.00^AB^
70% Dx	551.4 ± 2.9 ^AB^	660.4 ± 2.9 ^B^	742.0 ± 2.9 ^A^	1.20 ± 0.01 ^B^	1.35 ± 0.01 ^A^	1312.3 ± 1.8^A^	0.42 ± 0.00^C^
50% MDx	556.9 ± 5.5 ^B^	696.0 ± 5.0 ^C^	771.4 ± 6.2 ^AB^	1.25 ± 0.01 ^B^	1.39 ± 0.02 ^A^	1380.7 ± 1.3^E^	0.40 ± 0.00^A^
60% MDx	552.7 ± 7.5 ^AB^	662.8 ± 2.8 ^B^	746.2 ± 2.9 ^AB^	1.20 ± 0.02 ^B^	1.35 ± 0.01 ^A^	1359.6 ± 2.4^D^	0.40 ± 0.00^A^
70% MDx	541.7 ± 2.2 ^A^	598.8 ± 8.1 ^A^	738.5 ± 5.5 ^AB^	1.11 ± 0.01 ^A^	1.36 ± 0.01 ^AB^	1327.1 ± 1.1^B^	0.42 ± 0.01^BC^

Dx—tapioca dextrin used as a carrier, MDx—potato maltodextrin used as a carrier, T—inlet drying air temperature, n—rotary atomizer speed. ^a. b. c^ Homologous groups for one of the factors: T, *n*. ^A. B. C. D, E^ Homologous groups of factors: carrier type and content.

**Table 3:** Powder yield (Y), total polyphenol content (TPC) in solution before drying, total polyphenol content (TPC) and TPC retention (R_TPC_) in spray-dried chokeberry powder, trolox equivalent antioxidant capacity (TEAC) in solution before drying, trolox equivalent antioxidant capacity (TEAC) and TEAC retention (R_TEAC_) in spray-dried chokeberry powder.

Drying Conditions/Carrier Type and Content	Y (%)	TPC in Solution before Drying (mg GA/100 g d.m.)	TPC in Powder (mg GA/100 g d.m.)	R_TPC_ (%)	TEAC in Solution before Drying (mg TE/100 g d.m.)	TEAC in Powder (mg TE/100 g d.m.)	R_TEAC_ (%)
Dx, T = 150 °C	79.8 ± 1.9 ^a^	2080	2036 ± 17 ^c^	97.9 ± 0.8 ^c^	20.5	14.5 ± 0.5 ^b^	70.8 ± 2.4 ^b^
Dx, T = 160 °C	86.1 ± 2.6 ^ab^	2080	1756 ± 17 ^b^	84.4 ± 0.8 ^b^	20.5	17.1 ± 0.6 ^c^	83.5 ± 2.7 ^c^
Dx, T = 170 °C	88.9 ± 2.2 ^b^	2080	1643 ± 11 ^a^	79.0 ± 0.5 ^a^	20.5	10.1 ± 0.4 ^a^	49.3 ± 1.9 ^a^
Dx, *n* = 12,000 rpm	86.1 ± 2.6 ^a^	2080	1756 ± 17 ^a^	84.4 ± 0.8 ^a^	20.5	17.1 ± 0.6 ^a^	83.5 ± 2.7 ^a^
Dx, *n* = 13,500 rpm	84.8 ± 1.7 ^a^	2080	1826 ± 28 ^b^	87.8 ± 1.3 ^b^	20.5	19.2 ± 0.4 ^b^	94.1 ± 2.2 ^b^
Dx, *n* = 15,000 rpm	81.0 ± 2.0 ^a^	2080	2024 ± 7 ^c^	97.3 ± 0.3 ^c^	20.5	19.4 ± 0.3 ^b^	95.0 ± 1.5 ^b^
50% Dx	35.3 ± 4.1 ^B^	2600	2014 ± 11 ^E^	77.5 ± 0.4 ^C^	25.6	15.5 ± 0.5 ^D^	60.6 ± 1.9 ^B^
60% Dx	83.2 ± 1.9 ^D^	2080	1756 ± 17 ^C^	84.4 ± 0.8 ^D^	20.5	17.1 ± 0.6 ^E^	83.5 ± 2.7 ^D^
70% Dx	95.3 ± 1.6 ^E^	1560	1549 ± 25 ^B^	99.3 ± 1.6 ^E^	15.3	13.3 ± 0.3 ^C^	86.9 ± 2.1 ^D^
50% MDx	25.4 ± 3.9 ^A^	2600	1854 ± 14 ^D^	71.3 ± 0.5 ^A^	25.6	7.8 ± 0.3 ^A^	30.6 ± 1.1 ^A^
60% MDx	74.3 ± 1.6 ^C^	2080	1543 ± 6 ^B^	74.2 ± 0.3 ^B^	20.5	14.7 ± 0.4 ^D^	72.0 ± 2.1 ^C^
70% MDx	80.1 ± 2.7 ^CD^	1560	1309 ± 9 ^A^	83.9 ± 0.6 ^D^	15.3	11.6 ± 0.3 ^B^	75.5 ± 2.0 ^C^

Dx—tapioca dextrin used as a carrier, MDx—potato maltodextrin used as a carrier, T—inlet drying air temperature, *n*—rotary atomizer speed, ^a, b, c^ Homologous groups for one of the factors: T, *n*, ^A, B, C, D, E^ Homologous groups of factors: carrier type and content.
